# Virus-Induced Intestinal Barrier Injury: Mechanisms and Therapeutic Perspectives

**DOI:** 10.3390/vetsci13080764

**Published:** 2026-07-30

**Authors:** Huaming Xi, Jiacun Liu, Jing Wang, Li Zhong, Yigang Xu, Yuan Li

**Affiliations:** 1 Key Laboratory of Applied Biotechnology on Animal Science &Veterinary Medicine of Zhejiang Province, Zhejiang Engineering Research Center for Veterinary Diagnostics & Advanced Technology, Zhejiang International Science and Technology Cooperation Base for Veterinary Medicine and Health Management, Belt and Road International Joint Laboratory for One Health and Food Safety, China-Australia Joint Laboratory for Animal Health Big Data Analytics, College of Veterinary Medicine, Zhejiang A&F University, 666 Wusu Street, Lin’an District, Hangzhou 311300, China; 2 College of Animal Science & Technology, Zhongkai University of Agriculture and Engineering, Guangzhou 510550, China

**Keywords:** viral infection, intestinal barrier, immune response, molecular mechanisms, therapeutic strategies

## Abstract

The intestinal barrier is a key interface maintaining host–microbial segregation and systemic homeostasis. A broad range of viruses, including enteric, respiratory, and systemic pathogens, can disrupt this barrier through effects on epithelial integrity, vesicular transport, immune responses, and microbial ecology. Whether these diverse insults converge on shared regulatory nodes or act through distinct virus-specific pathways that ultimately result in barrier failure remains unclear. Building on this premise, this review systematically delineates the molecular and cellular mechanisms underlying virus-induced disruption of the intestinal barrier. Viral infection disrupts epithelial integrity through multiple converging processes, including disassembly of tight junction architecture, activation of programmed cell death pathways, degradation of the mucus layer, impaired regeneration driven by intestinal stem cells, and dysregulation of transcellular transport. These processes are interconnected and collectively drive epithelial dysfunction and barrier breakdown. Beyond epithelial damage, we further highlight the pivotal contribution of host immune responses to barrier breakdown. Viral infection induces dysregulated cytokine production and aberrant immune activation, which amplify epithelial damage and further increase barrier permeability. In parallel, increasing evidence supports a bidirectional interaction between viral infection and gut microbiota dysbiosis, in which each process reinforces the other to accelerate barrier disruption and disease progression. We also discuss emerging therapeutic strategies aimed at restoring intestinal homeostasis, including antiviral therapies, host-targeted interventions, and microbiota modulation. Despite recent progress, key questions remain, particularly regarding mechanisms of failed barrier repair after viral clearance and the multilayered regulatory networks linking viruses, immunity, and the microbiota. Together, this review provides a framework for understanding virus-induced intestinal barrier dysfunction and identifies potential therapeutic nodes for intervention.

## Simple Summary

Viral infections often lead to severe gastrointestinal symptoms, yet the exact ways these viruses damage the gut remain complex. The intestinal barrier acts as a crucial defense line, consisting of cell connections, a protective mucus layer, and beneficial gut bacteria. In this article, we comprehensively explore how various viruses—such as Rotavirus and PEDV—disrupt this vital barrier. We detail how these pathogens dismantle the tight structures holding intestinal cells together and trigger different forms of cell death. Furthermore, we explain how viral infections thin the protective mucus layer and cause an imbalance in the gut microbiome (dysbiosis), leading to increased inflammation and weakened immune defenses. By summarizing these diverse viral strategies, this paper provides a clear picture of how viruses cause intestinal damage. Understanding these underlying processes is highly significant, as it highlights potential new targets for developing effective therapies and dietary interventions to restore gut health and combat viral gastrointestinal diseases.

## 1. Introduction

The intestinal barrier is a highly specialized, multi-layered interface that maintains a fundamental biological paradox: enabling nutrient absorption while simultaneously restricting the systemic dissemination of luminal microorganisms and antigens [1]. This dynamic equilibrium underpins host metabolic homeostasis, immune tolerance, and resistance to infection. Structurally and functionally, the barrier integrates epithelial integrity, mucus-mediated chemical defense, mucosal immunity, and the gut microbiota into a coordinated system of surveillance and protection [2]. Disruption of this integrated network is increasingly recognized as a central driver of local and systemic disease.

Historically, intestinal barrier dysfunction has been primarily attributed to bacterial pathogens and chronic inflammatory disorder [3,4,5]. However, a growing body of evidence now implicates viruses as important and mechanistically distinct perturbators of barrier integrity [6]. Recent studies have fundamentally reshaped the understanding of virus–gut interactions, demonstrating that intestinal barrier dysfunction is not restricted to classical enteric infections but represents a common pathological feature associated with diverse systemic viral infections [7,8,9]. These findings raise a critical unresolved question: whether different viruses disrupt intestinal barrier integrity through virus-specific mechanisms or converge on shared host regulatory pathways. Despite substantial progress, the mechanistic principles governing virus-induced barrier collapse remain poorly defined. In particular, it remains unclear how epithelial injury, immune activation, metabolic remodeling, and microbiota dysbiosis are temporally and functionally interconnected, and whether conserved host regulatory nodes determine barrier failure across distinct viral infections. Emerging studies suggest that virus-induced barrier dysfunction is not a consequence of isolated epithelial damage, but rather the result of coordinated disruption across multiple biological layers. Viral infection simultaneously targets tight junction (TJ) architecture, epithelial cell survival, mucus production, immune homeostasis, and microbial ecology [10]. These processes are not independent; instead, they form a self-reinforcing network in which epithelial injury, immune activation, and dysbiosis amplify one another, ultimately driving barrier collapse and systemic disease progression [11]. Despite these advances, a unifying mechanistic framework remains lacking.

Addressing these gaps is not only of fundamental biological importance but also of substantial clinical relevance. Intestinal barrier dysfunction has emerged as a critical determinant of disease severity in viral infections, contributing to systemic inflammation, multi-organ complications, and long-term sequelae. Importantly, recent advances in multi-omics, spatial biology, and host–microbiota interaction studies have shifted the field from identifying individual viral effects toward understanding shared host mechanisms that govern barrier resilience and failure. This conceptual transition highlights the need for an integrated framework that connects viral infection, barrier disruption, and systemic pathology. Yet, current therapeutic strategies largely focus on viral suppression and often fail to restore barrier integrity or mucosal homeostasis. In this review, we synthesize current knowledge on virus-induced intestinal barrier dysfunction through an integrative lens, spanning epithelial biology, immunology, and host–microbiota interactions. We propose a conceptual framework in which diverse viruses, despite differences in tropism and replication strategies, converge on common host regulatory axes involving epithelial junction remodeling, cell death pathways, immune–metabolic reprogramming, and microbiota imbalance to drive intestinal barrier failure. We delineate the molecular and cellular mechanisms by which viruses disrupt barrier function, examine the bidirectional interplay between viral infection and the gut ecosystem, and evaluate emerging therapeutic strategies aimed at restoring barrier integrity. By framing these processes within a unified conceptual model, we aim to identify key regulatory nodes and highlight critical knowledge gaps that will guide future research and therapeutic innovation.

## 2. The Intestinal Barrier as an Integrated Defense System

**Figure 1:**
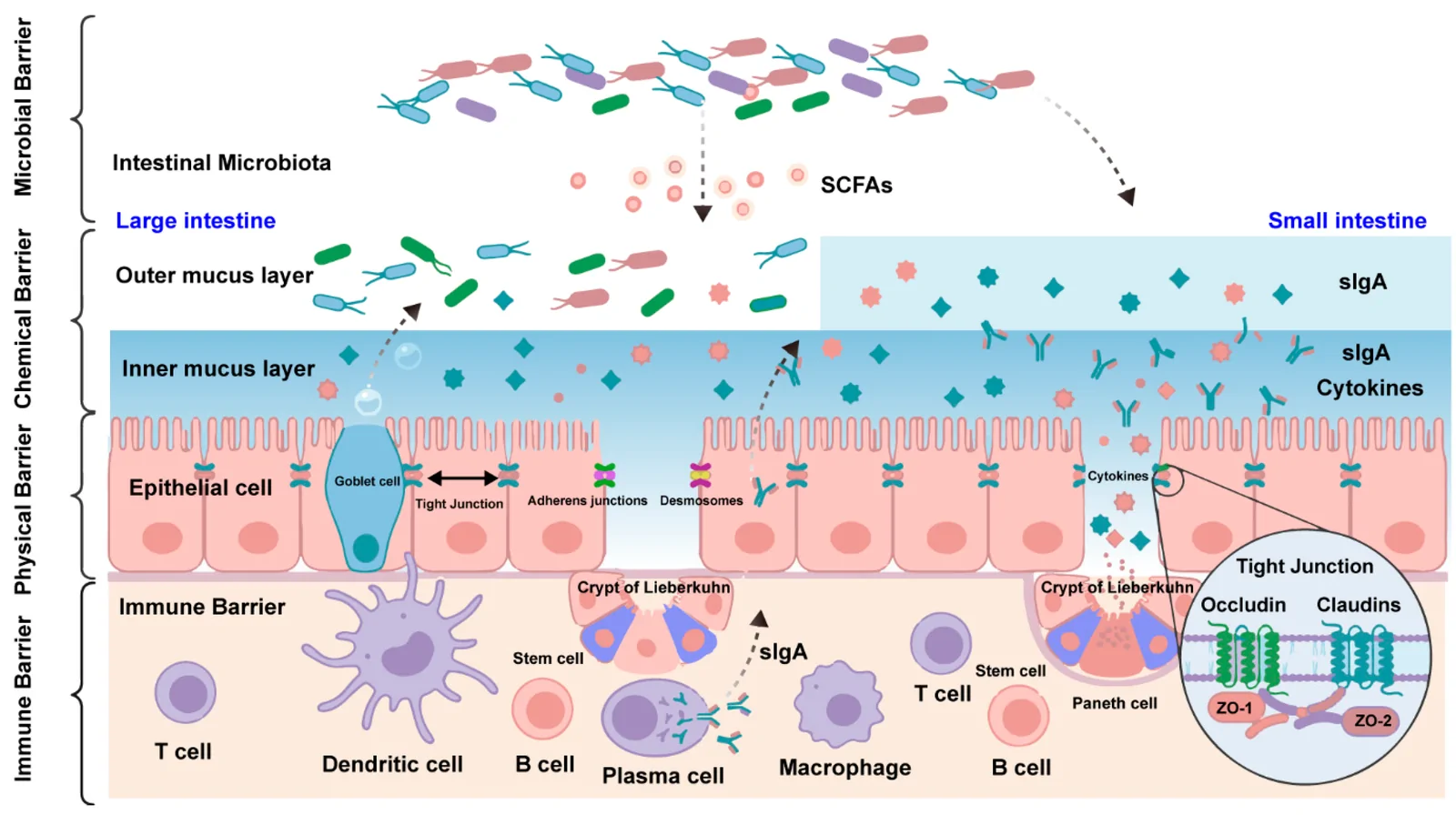
The intestinal barrier consists of four interconnected functional compartments: the microbial, chemical, physical, and immune barriers, which collectively maintain intestinal homeostasis and prevent microbial invasion. The microbial barrier is primarily composed of intestinal microbiota, which are more abundant in the large intestine and produce metabolites such as SCFAs that support epithelial integrity and immune homeostasis. The chemical barrier is formed by the mucus layer and antimicrobial factors. The large intestine contains a well-developed two-layer mucus structure, including an outer loose mucus layer and an inner dense mucus layer, whereas the small intestine relies more on sIgA, antimicrobial molecules, and cytokines for microbial control and mucosal defens. The physical barrier is maintained by intestinal epithelial cells, including goblet cells and absorptive epithelial cells, which are interconnected through junctional complexes, including tight junctions (occludin, claudins, ZO-1, and ZO-2), adherens junctions, and desmosomes. Intestinal epithelial renewal is supported by stem cells located within the Crypts of Lieberkühn, which exhibit distinct regional characteristics between the small intestine and large intestine. Small intestinal crypts contain abundant Paneth cells at the crypt base, which secrete antimicrobial peptides and maintain the stem cell niche, whereas large intestinal crypts generally lack typical Paneth cells and are enriched in goblet cells, colonocytes, and intestinal stem cells, supporting mucus production, epithelial renewal, and adaptation to the dense microbial environment. The immune barrier contains dendritic cells, macrophages, T cells, B cells, and plasma cells, which regulate microbial composition and mucosal immunity through immune surveillance and sIgA production.

The intestinal barrier is a multi-component, hierarchical defense system that isolates the host’s internal tissues from the complex intestinal lumen environment containing microorganisms, food antigens, and various exogenous substances, and its structural and functional integrity is the core of maintaining intestinal homeostasis [12]. As shown in Figure 1, this barrier consists of physical, chemical, immune, and microbial barriers, and abnormal function of any component can lead to a decrease in overall defense capacity [13].

### 2.1. Physical Barrier

The physical barrier is the core structure of the intestinal barrier, which is composed of a single layer of intestinal epithelial cells and tight connections between adjacent epithelial cells, and the polar distribution of epithelial cells and intercellular adhesion junctions also provide structural support for the physical barrier [14]. Morphologically and functionally, the physical barrier differs between these regions: the small intestine features villi and crypts of Lieberkühn, containing stem cells that divide continuously at the base of the crypts and intercalate with Paneth cells (which secrete antimicrobial peptides), alongside relatively more permeable TJs to maximize nutrient absorption [15]. The epithelial junctional complex is organized in a characteristic apical-to-basal arrangement consisting of TJs (TJs; zonula occludens), adherens junctions (AJs; zonula adherens), and desmosomes (macula adherens). These three junctional structures function cooperatively to preserve epithelial architecture and resist mechanical and microbial insults. Among these junctional complexes, TJs play a central role in maintaining epithelial integrity. TJs are multi-protein assemblies composed of transmembrane proteins (such as claudins and occludin), cytoplasmic scaffold proteins (such as ZO proteins), and cytoskeletal elements, which together form a dynamic sealing network between adjacent epithelial cells [16]. This structure strictly regulates paracellular transport, allowing controlled passage of ions and small solutes while effectively preventing the translocation of luminal pathogens, toxins, large macromolecules and microbiota. In addition to serving as a passive barrier, TJs are highly dynamic and responsive to physiological and pathological stimuli, making them a critical regulatory hub in maintaining intestinal homeostasis. To establish systemic infection, viruses must first traverse the mucosal epithelium and subsequently disseminate throughout the host. However, mucosal epithelial layers are reinforced by well-developed TJs that serve as a critical barrier against the spread and dissemination of viral pathogens. Viruses can bypass this barrier by disrupting epithelial TJs, thereby promoting paracellular entry and enabling systemic infection.

### 2.2. Chemical Barrier

The chemical barrier constitutes an essential protective layer overlaying the intestinal epithelium, primarily composed of the mucus layer and a variety of antimicrobial molecules. The mucus layer is predominantly built upon mucin MUC2, which is secreted by goblet cells and serves as the structural backbone of this barrier [17]. Functionally, the mucus layer is organized into two distinct compartments: a dense, inner layer that is firmly attached to the epithelium and largely sterile, and a looser, outer layer that harbors commensal microorganisms. This spatial organization not only physically prevents direct contact between pathogens and epithelial cells but also contributes to immune signaling regulation, epithelial repair, and cell proliferation [18]. In addition to mucus, antimicrobial peptides (AMPs) represent another key effector of the chemical barrier. These peptides are secreted primarily by Paneth cells and, to a lesser extent, by intestinal epithelial cells. AMPs exhibit broad-spectrum antimicrobial activity against bacteria, fungi, and viruses by disrupting microbial membranes or interfering with essential microbial processes [19]. Many enteric viruses can impair mucus production by affecting goblet cell function or altering mucin expression and secretion, resulting in thinning or disorganization of the mucus layer. This weakens the separation between luminal pathogens and epithelial cells, facilitating their contact with the epithelial surface. Viruses may also modulate antimicrobial peptides, such as those secreted by Paneth cells, further compromising local antimicrobial defense. Collectively, these effects impair the intestinal chemical barrier and increase susceptibility to infection and inflammation.

### 2.3. Immune Barrier

The immune barrier serves as the central immunological defense system of the intestinal mucosa, integrating both innate and adaptive immune components. It is mainly composed of gut-associated lymphoid tissue (GALT), a diverse population of immune cells residing in the lamina propria (including macrophages, dendritic cells, T cells, and B cells), as well as secretory immunoglobulin A (sIgA) [20]. Among these components, sIgA is a key effector molecule of mucosal immunity. It is produced by plasma cells in the lamina propria and transported across the epithelium into the intestinal lumen, where it binds to pathogens, toxins, and antigens. By neutralizing these luminal factors and preventing their adhesion to epithelial cells, sIgA provides a first line of immune exclusion without triggering excessive inflammation [21]. Meanwhile, antigen-presenting cells such as macrophages and dendritic cells continuously sample luminal contents and recognize pathogen-associated molecular patterns (PAMPs). Upon activation, they initiate innate immune responses and coordinate the activation of adaptive immunity, leading to targeted pathogen clearance and immune memory formation [22]. Through these tightly regulated processes, the immune barrier not only eliminates invading pathogens but also maintains immune tolerance to commensal microbiota, thereby preserving intestinal immune homeostasis. However, under viral infection, this balance can be disrupted. Viruses disrupt the intestinal immune barrier by impairing immune cell function, dysregulating cytokine responses, and reducing secretory IgA-mediated mucosal defense, ultimately weakening local immune homeostasis and facilitating infection.

### 2.4. Microbial Barrier

The microbial barrier is formed by the dense and diverse community of symbiotic microorganisms colonizing the intestinal tract, collectively known as the gut microbiota. Importantly, the distribution and composition of this microbiota differ significantly between the small and large intestines, driven by major physiological and environmental factors. The small intestine harbors a relatively lower density of bacteria due to restrictive factors such as rapid luminal transit time, lower pH, and high concentrations of bile acids and antimicrobial peptides. In contrast, the large intestine contains a highly dense and diverse microbial community, primarily dominated by *Firmicutes* and *Bacteroidetes*, facilitated by slower transit times, a more neutral pH, and abundant fermentable substrates [23]. This microbial ecosystem contributes to intestinal barrier homeostasis through multiple mechanisms, including nutrient competition, occupation of ecological niches, and production of antimicrobial metabolites such as short-chain fatty acids (SCFAs), which collectively suppress the overgrowth of pathogenic bacteria [24]. Beyond colonization resistance, the gut microbiota plays a crucial regulatory role in host physiology. It influences intestinal epithelial cell proliferation and differentiation, strengthens epithelial integrity, and modulates the development and maturation of the mucosal immune system. Through these interactions, the microbiota establishes a functional “microecological shield” that is indispensable for maintaining intestinal barrier stability [25]. Importantly, disruption of this microbial equilibrium (dysbiosis) is closely associated with impaired barrier function and increased susceptibility to disease. Although intestinal barrier dysfunction has traditionally been associated with bacterial infections, inflammatory bowel diseases, and dietary disturbances, increasing evidence suggests that viruses also play an important role [26]. Viruses disrupt the intestinal microbial barrier by inducing gut dysbiosis, altering microbial composition and function, and weakening colonization resistance, thereby exacerbating epithelial injury and inflammatory responses.

## 3. Direct Epithelial Targeting as the Initiating Event of Barrier Failure

The intestinal physical barrier is a highly organized epithelial structure that maintains mucosal integrity and prevents pathogen dissemination. Direct damage to the intestinal barrier by viruses is an important initiator of barrier dysfunction, which is mainly achieved by destroying TJ structures, inducing epithelial cell death, and impairing intestinal stem cell function. These processes often occur in a coordinated manner and are further amplified by virus-induced inflammatory responses and mechanical stress within the epithelial layer. Crucially, the exact mechanisms of barrier failure must be categorized by viral behavior, delineating between barrier disruption driven by luminal entry and primary enterocyte infection, and disruption driven by vascular entry and lamina propria immune activation.

### 3.1. Disruption of Tight Junction

**Figure 2:**
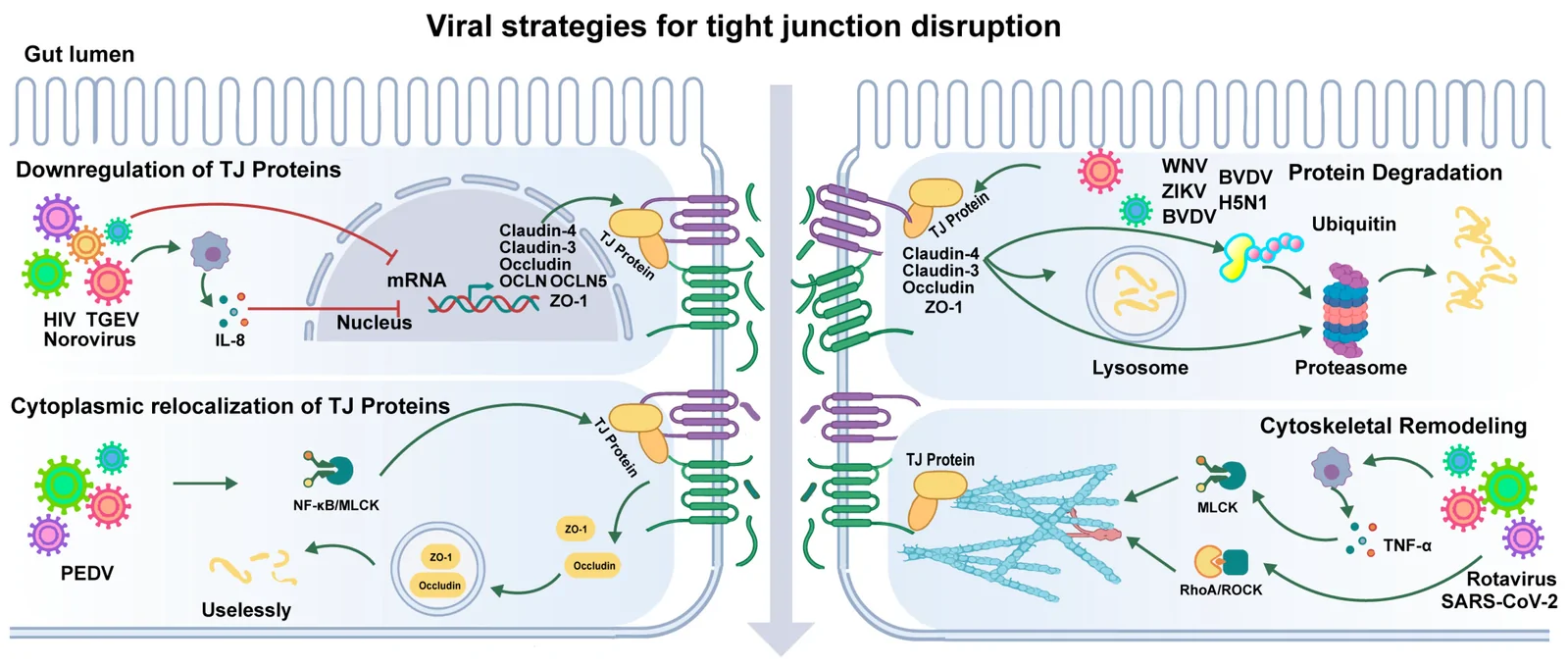
Viral strategies for tight junction (TJ) disruption. This schematic summarizes four primary mechanisms by which pathogenic viruses compromise epithelial barrier integrity: downregulation of TJ proteins through the inhibition of mRNA transcription (e.g., HIV, Norovirus); direct protein degradation via lysosomal or ubiquitin-proteasome pathways (e.g., ZIKV, H5N1); cytoplasmic relocalization, causing membrane TJ proteins to internalize and lose their barrier function (e.g., PEDV); and cytoskeletal remodeling, where inflammatory cytokines activate downstream signaling (such as MLCK or RhoA/ROCK) to physically disrupt the junction structure (e.g., Rotavirus, SARS-CoV-2).

TJs constitute the structural core of the intestinal epithelial barrier and are essential for maintaining paracellular integrity [27,28]. Disruption of TJs is widely recognized as a key event in virus-induced barrier leakage [29]. A broad range of viruses target TJ complexes composed of claudins, occludin, and ZO proteins, resulting in altered expression, mislocalization, and degradation [30,31,32,33]. Mechanistically, viruses regulate TJ integrity through multiple coordinated processes, including transcriptional suppression of TJ components, proteolytic degradation, redistribution of junctional proteins, and cytoskeletal remodeling (Figure 2). This multi-layered assault effectively compromises epithelial and endothelial barrier integrity, facilitating viral dissemination and pathogenesis. The specific viral factors, host targets, and combined mechanisms utilized by diverse viruses are comprehensively summarized in Table 1. However, a critical appraisal is necessary: much of the foundational data detailing these molecular mechanisms is derived from immortalized epithelial monolayers (e.g., Caco-2, MDCK, or IPEC-J2 cell lines). These simplistic in vitro systems inherently lack the complex stromal, immune, and microbial cross-talk of a live host, necessitating a careful synthesis of how these mechanisms actually translate in vivo.

#### 3.1.1. Transcriptional Repression and Proteolytic Degradation of TJ Components

At the transcriptional level, diverse viruses suppress key TJ components, thereby compromising epithelial and endothelial barrier integrity. For strictly enteric viruses initiating infection from the lumen, transmissible gastroenteritis virus (TGEV) suppresses the expression of Occludin and ZO-1 [34]. Parallel to Occludin/ZO-1 disruption, the Claudin family is systematically targeted to collapse barrier structure. TGEV and Norovirus reduce the levels of claudin-3 and claudin-4, respectively. Demonstrating a multifaceted viral strategy, Norovirus couples the transcriptional downregulation of claudin-4 with direct structural perturbations of epithelial adhesion mediated by its capsid protein VP1 [35,36]. Conversely, for systemic viruses invading from the basolateral compartment, at the transcriptional level, Human Immunodeficiency Virus (HIV) (via the viral protein Tat) suppresses the expression of Occludin, ZO-1, and CLDN5 [37]. This virus-driven repression is further amplified by host inflammatory mediators, such as CXCL8, which broadly reduce the expression of these proteins [38]. At the post-translational level, other viruses rapidly clear pre-existing TJ components by hijacking host ubiquitin-proteasomal pathways. For instance, both bovine viral diarrhea virus (BVDV) NS5A and H5N1 influenza virus specifically accelerate occludin turnover; notably, H5N1 achieves this by activating the E3 ubiquitin ligase Itch, reinforcing rapid TJ disruption [39,40]. Although hijacking host ubiquitination is common, the functional hierarchy of the involved E3 ubiquitin ligases (e.g., Itch, NEDD4 family, SCF complexes) remains unresolved. Identifying whether distinct viruses converge on central ubiquitination hubs or deploy virus-specific degradation circuits is critical for understanding proteostatic control during infection. Crucially, the manuscript must explicitly state that these transcriptional suppression and proteolytic clearance pathways have primarily been proven in vitro. Accepting these findings at face value obscures the reality of the live host. In vivo, lamina propria immune responses can profoundly alter these observed TJ disruptions. While a virus may directly initiate modest TJ degradation in a monolayer, the in vivo recruitment of macrophages and neutrophils, and their subsequent release of massive cytokine storms (e.g., IFN-γ, IL-1β), can aggressively amplify this breakdown, often shifting the primary driver of barrier failure from direct viral cytopathology to host immunopathology.

#### 3.1.2. Protein Redistribution and Cytoskeletal Remodeling

Beyond proteolytic degradation, viruses can compromise TJ integrity by driving the mislocalization of junctional proteins from the plasma membrane to the cytoplasm, thereby precipitating functional disassembly of epithelial barriers. During direct luminal infection, strictly enteric viruses deploy simultaneous strategies to physically tear junctions apart. Rotavirus, for example, exhibits a highly coordinated structural assault: its non-structural proteins NSP4 and NSP1 induce the direct cytoplasmic relocalization of ZO-1 and occludin, respectively [41], while it concurrently activates the RhoA/ROCK signaling pathway to drive actin cytoskeleton remodeling, profoundly exacerbating TJ disruption [42]. Furthermore, the myosin light chain kinase (MLCK) pathway emerges as a convergent functional node exploited by diverse viruses to drive both spatial and cytoskeletal perturbations. In the case of porcine epidemic diarrhea virus (PEDV), engagement of epithelial receptors by the spike (S) protein activates NF-κB/MLCK signaling, which directly promotes the redistribution of ZO-1 and occludin away from the junctional complex [43,44]. Systemic viruses utilize parallel mechanical routes; SARS-CoV-2 targets epithelial integrity through ACE2-mediated signaling to activate the MLCK pathway, promoting myosin light chain phosphorylation, robust cytoskeletal contraction, and the physical expansion of intercellular spaces [45]. In parallel, host inflammatory signaling provides a potent reinforcing layer to this mechanical stress. For a veterinary audience, African Swine Fever Virus (ASFV) serves as a critical model for this vascular-driven barrier collapse. By severely targeting macrophages and monocytes in the lamina propria rather than causing direct enterocyte necrosis, ASFV induces a massive cytokine storm [46]. Tumor necrosis factor-α (TNF-α), for instance, directly induces MLCK expression, further enhancing epithelial contractility and increasing TJ permeability. However, it is still unclear whether viral cues engage cytoskeletal regulators through the direct modulation of Rho GTPases or via intermediary kinases (such as Src, PKC, or PI3K). Likewise, whether diverse viruses converge on a unified RhoA/ROCK–MLCK axis or instead exploit virus-specific routes that ultimately produce convergent junctional phenotypes remains to be resolved.

**Table 1:** Destruction of TJs by virus.

Virus	Viral Factor	Host Target(s)	Integrated Mechanisms/Pathways	References
Rotavirus	NSP4, NSP1	ZO-1, Occludin, Actin	Protein redistribution (cytoplasmic relocalization); cytoskeletal remodeling (RhoA/ROCK pathway)	[28,42]
PEDV	Spike (S) protein	ZO-1, Occludin	Protein redistribution (via NF-κB/MLCK signaling); transcriptional alterations	[43,44]
Norovirus	VP1 capsid	Claudin-4, Adhesion complexes	Transcriptional downregulation; direct structural perturbation	[35,36]
TGEV	Not specified	Claudin-3, Occludin	Transcriptional suppression	[34]
HIV	Tat	OCLN, CLDN5, ZO-1	Transcriptional suppression	[37]
SARS-CoV-2	Not specified	Cytoskeleton (via ACE2)	Cytoskeletal remodeling (MLCK pathway activation, contraction)	[45]
H5N1 Influenza	Not specified	Occludin	Proteolytic degradation (ubiquitin-dependent via Itch E3 ligase)	[40]
BVDV	NS5A	Occludin	Proteolytic degradation (ubiquitin-mediated)	[39,40]
ASFV	Not specified	Occludin	MLCK expression	[46]

### 3.2. Induction of Intestinal Epithelial Cell Death

**Figure 3:**
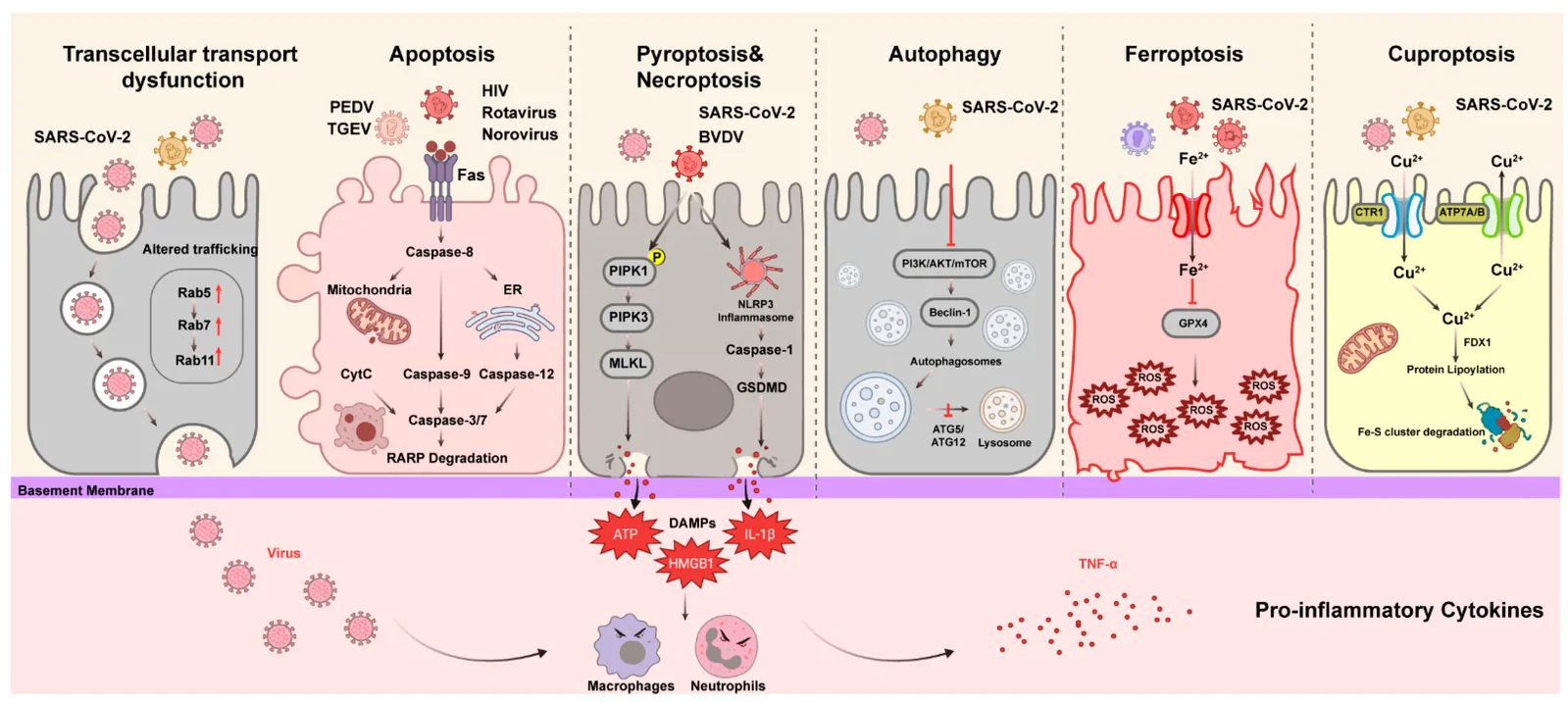
Mechanism of virus-induced intestinal epithelial cell dysfunction and death. Viral infection triggers transcellular transport dysfunction, apoptosis, pyroptosis, necroptosis, autophagy, ferroptosis, and cuproptosis in intestinal epithelial cells. Apoptosis, induced by viruses such as PEDV, TGEV, HIV, Rotavirus, and Norovirus, involves Fas–caspase and mitochondrial cytochrome c signaling. Pyroptosis and necroptosis, triggered by SARS-CoV-2 and BVDV, act via inflammasomes and RIPK1/RIPK3/MLKL, causing membrane rupture and the release of IL-1β, ATP, and HMGB1. Autophagy, modulated by SARS-CoV-2, is mediated by PI3K/AKT/mTOR and ATG pathways. Ferroptosis (induced by SARS-CoV-2) results from GPX4 inhibition and iron-dependent lipid peroxidation, while cuproptosis (also triggered by SARS-CoV-2) arises from copper-induced mitochondrial dysfunction. Together, these pathways—along with SARS-CoV-2-induced transcellular transport dysfunction—amplify intestinal inflammation, recruit immune cells (macrophages and neutrophils), and enhance pro-inflammatory cytokine production (e.g., TNF-α).

Intestinal epithelial cells form the structural and functional basis of the intestinal barrier and work together with TJ complexes to maintain selective permeability [47]. As discussed above, viruses disrupt TJ integrity through transcriptional suppression, protein degradation, junctional mislocalization, and cytoskeletal remodeling, but these changes alone do not fully explain the extent of barrier failure observed in vivo. Viral infection therefore extends beyond junctional disruption to affect the epithelial cell layer itself. In this setting, viruses impair epithelial integrity mainly through two linked processes, altered transcellular transport and programmed cell death (Figure 3), which together increase epithelial permeability and drive progressive barrier breakdown.

#### 3.2.1. Transcellular Transport Dysfunction

Transcellular transport, including endocytosis and transcytosis, is a key route for material exchange across the intestinal epithelium. Under normal conditions, endocytosis internalizes luminal contents, whereas transcytosis mediates their directed transport to the basolateral surface, supporting nutrient absorption while limiting the passage of harmful substance [48,49]. During primary luminal infections, viral infection disrupts this system by altering vesicle trafficking and membrane dynamics. Viral surface proteins bind epithelial receptors and activate endocytic pathways, such as clathrin- or caveolin-mediated uptake, and then utilize intracellular transport machinery to promote entry and spread [50,51]. As a result, viruses can spread locally within the intestinal mucosa while concurrently increasing epithelial permeability, allowing pathogenic bacteria and toxins to enter the circulation, amplify systemic inflammation, and indirectly compromise barrier function [52,53,54]. This may involve changes in Rab GTPase-regulated trafficking (including Rab5, Rab7 and Rab11), endosomal maturation and recycling, and sorting processes involving ESCRT complexes, although the underlying mechanisms remain unclear. It is also not known whether viruses selectively engage specific endocytic adaptors (such as AP2 or dynamin) or depend on particular ubiquitin signals to direct cargo sorting. These alterations increase transcellular permeability, allowing luminal bacteria and toxins to enter the circulation and amplify systemic inflammation. Conversely, representing a systemic route of entry, SARS-CoV-2 provides an example of this process by linking vesicle trafficking changes with intercellular spread and barrier dysfunction [55].

#### 3.2.2. Programmed Cell Death

As discussed above, disruption of TJs weakens paracellular sealing, but does not fully account for barrier failure; viral infection further targets epithelial cell function, extending injury to transcellular transport and polarity, thereby driving more extensive barrier breakdown.

(1) Apoptosis

During viral infection, apoptosis fundamentally serves as a natural host defense mechanism aimed at restricting viral dissemination; however, the induction of apoptosis in intestinal epithelial cells inevitably leads to increased damage to the intestinal barrier. Apoptosis is predominantly mediated through the coordinated activation of extrinsic (death receptor–dependent) and intrinsic (mitochondria-dependent) caspase cascades [56]. Although the downstream execution pathways are largely conserved, different viruses engage these networks through distinct upstream cues, often converging on a similar dual-pathway outcome while leaving key initiating mechanisms unresolved. In luminal infections, direct enterocyte apoptosis is prominent. Rotavirus and norovirus both activate mixed apoptotic programs: rotavirus NSP4 triggers caspase-8-dependent signaling alongside mitochondrial depolarization [57], while norovirus NS3 induces caspase-8 activation and mitochondrial permeabilization [58]. Similarly, TGEV induces apoptosis through both the death receptor and mitochondrial pathways [59]. PEDV activates caspase-3/7 via its nucleocapsid protein, leading to PARP cleavage and TJ disruption [60]. In both TGEV and PEDV infections, whether junctional injury is a direct viral effect or a downstream consequence of apoptotic signaling remains uncertain. HIV induces intestinal epithelial apoptosis via Tat-mediated activation of caspase-9 and caspase-3 [61]. By contrast, during systemic disruptions, SARS-CoV-2 promotes apoptosis through caspase-8/caspase-3 activation following PI3K/Akt suppression [62], and CPV-2 simultaneously engages Fas/FasL-mediated extrinsic apoptosis and ER stress-associated caspase-12 activation [63].

(2) Pyroptosis and necroptosis

Beyond non-inflammatory apoptosis, highly inflammatory PCD pathways—namely necroptosis and pyroptosis—are also key contributors to barrier disruption (Figure 3), particularly in systemic infections. SARS-CoV-2 activates both necroptosis via RIPK3/MLKL and pyroptosis through NLRP3 inflammasome–caspase-1 signaling [64,65], leading to severe epithelial rupture and strong cytokine amplification. Similarly, BVDV induces necroptosis via NS4B-mediated engagement of RIPK1 and downstream RIPK3/MLKL activation [39,40]. Yet, the structural basis of NS4B interaction with host death complexes, and whether additional host cofactors are required to specify necroptotic signaling, remain undefined.

(3) Autophagy

Autophagy is a conserved homeostatic pathway that degrades damaged organelles and intracellular pathogens, but is frequently subverted by viruses to support replication or trigger autophagy-associated cell death, thereby aggravating epithelial injury and barrier dysfunction (Figure 3) [66]. Diverse viruses frequently converge on a shared strategy: initiating autophagosome formation while deliberately blocking terminal lysosomal degradation to induce severe autophagic stress. Following luminal entry, Norovirus initiates this by suppressing the PI3K/AKT/mTOR signaling axis and simultaneously targeting the ATG5–ATG12 complex to block autophagosome–lysosome fusion, leading to organelle accumulation and secondary TJ downregulation [67,68]. In a systemic infection model, SARS-CoV-2 utilizes its ORF3a protein to interact with Beclin-1, aggressively promoting autophagosome biogenesis while independently impairing lysosomal degradation [69,70]. By uncoupling autophagosome formation from lysosomal fusion, these viruses transform a vital host survival mechanism into a lethal driver of epithelial injury.

(4) Metal-dependent cell death: ferroptosis and cuproptosis

These metabolic death pathways are predominantly characterized in systemic infections. Ferroptosis is an iron-dependent form of regulated cell death driven by lipid peroxidation and characterized by ROS accumulation, iron overload, and GPX4 inactivation [71]. Increasing evidence indicates that it contributes to virus-induced intestinal epithelial injury by linking redox imbalance with barrier breakdown [72]. During SARS-CoV-2 infection, epithelial cells exhibit suppressed GPX4 expression and enhanced iron uptake, shifting the redox balance toward lipid peroxide accumulation—a process further amplified by TNF-α-mediated ROS generation [73,74]. Similarly, influenza virus induces intestinal ferroptotic injury via its circulating neuraminidase (NA), which degrades epithelial surface sialic acids to facilitate iron influx and subsequently reduces GPX4 expression [75,76].

Parallel to iron dysregulation, viral infections can trigger cuproptosis, a recently identified modality driven by intracellular copper accumulation. Mechanistically, excess copper binds lipoylated enzymes of the tricarboxylic acid (TCA) cycle, causing the loss of iron–sulfur cluster proteins and leading to mitochondrial proteotoxic stress (Figure 3) [77]. In SARS-CoV-2-infected intestinal epithelial cells, this copper imbalance is induced by increased CTR1-mediated uptake and reduced ATP7A/ATP7B efflux. This intracellular copper surge promotes FDX1-dependent protein lipoacylation and aberrant copper–protein interactions, ultimately resulting in metabolic collapse and cuproptotic cell death [78,79].

Above all, virus-induced cell death pathways function not as isolated mechanisms, but as a highly interconnected network, exemplified by PANoptosis—an integrated complex encompassing pyroptosis, apoptosis, and necroptosis. At this network’s core, caspase-8 acts as a master switch: while it directly executes apoptosis, its viral suppression relieves inhibition on RIPK3 to drive necroptosis. Concurrently, active caspase-8 can cleave GSDMD to initiate pyroptosis. This axis intimately intersects with autophagic and metal-dependent stress. Virus-induced autophagic flux blockage and ferroptotic/cuproptotic metabolic collapse generate excessive ROS and damage-associated molecular patterns (DAMPs). These signals act as potent triggers for NLRP3 inflammasome activation (pyroptosis) and amplify inflammatory death cascades in neighboring cells. Ultimately, epithelial barrier disruption is driven by the dynamic cross-regulation of these integrated molecular hubs rather than any single, isolated mechanism.

### 3.3. Impairment of the Intestinal Stem Cells

**Figure 4:**
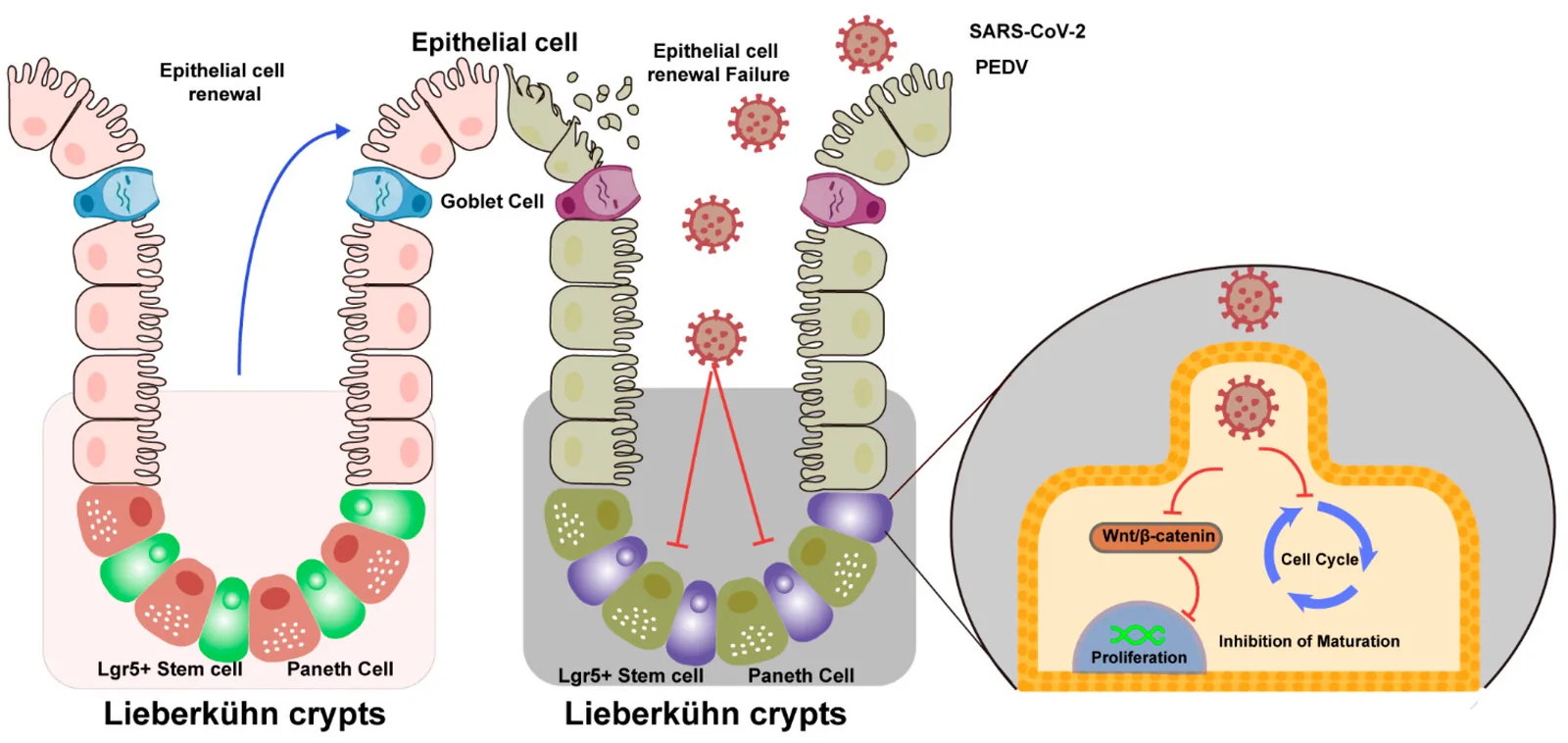
Viral infection of intestinal stem cells and impaired regenerative function. The normal intestinal Lieberkühn crypts, where Lgr5^+^ stem cells and Paneth cells regulate epithelial cell renewal via Wnt/β-catenin signaling, promoting proliferation and goblet cell differentiation while inhibiting maturation. Viral infection disrupts this Lieberkühn crypts, leading to impaired regenerative capacity of the intestinal epithelium.

Intestinal stem cells (ISCs), located at the base of intestinal crypts, drive continuous epithelial renewal by generating absorptive and secretory lineages, thereby maintaining mucosal homeostasis and barrier integrity [80,81]. Viral infections impair this regenerative capacity through two distinct mechanisms: the direct viral infection of Lgr5+ crypt stem cells, and indirect regenerative failure secondary to the inflammatory destruction of the supportive crypts of Lieberkühn, which includes Paneth cells and the surrounding mesenchyme. Both pathways lead to defective epithelial renewal and sustained barrier dysfunction (Figure 4).

Regarding direct cellular targeting, some strictly enteric viruses exhibiting luminal injury, such as Rotavirus, directly infect Lgr5+ ISCs. This infection induces cell cycle arrest, blocking differentiation into mature epithelial cells and inherently impairing barrier restoration [82,83]. Yet the upstream checkpoints controlling this arrest are undefined, including whether p53, RB, or DNA damage-like responses are involved, and how viral proteins interface with ISC-specific cell cycle machinery. Similarly, for systemic viruses driving basolateral injury, SARS-CoV-2 directly targets Lgr5+ ISCs via the ACE2 receptor, suppressing Wnt/β-catenin signaling to reduce proliferative capacity and delay epithelial turnover [84].

Conversely, regenerative failure frequently occurs secondary to niche compromise. Animal coronaviruses such as PEDV suppress ISC proliferation and differentiation, causing mucosal atrophy and severe barrier failure in piglets [85]. TGEV similarly downregulates Wnt signaling and disrupts crypt–villus architecture. While these viruses can interact with ISCs, a major driver of this regenerative failure is the virus-induced inflammatory destruction of supportive Paneth cells and the underlying mesenchymal niche. The loss of critical niche-derived survival and differentiation signals fundamentally limits ISC function. In PEDV, the exact balance between direct Lgr5+ cell infection and secondary niche destruction remains unresolved, as does the question of which molecular mediators (e.g., Notch, BMP, or inflammatory cytokines) dominate this secondary signaling collapse [86]. Collectively, these findings identify ISC impairment as a central mechanism of virus-induced failure of epithelial regeneration. However, key questions remain, including whether Lgr5+ ISCs serve as permissive viral reservoirs or transiently infected bystander cells, how the Paneth cell and mesenchymal niche is structurally and functionally remodeled during infection, and whether virus-induced stem cell dysfunction is reversible or leads to long-term reprogramming of epithelial regenerative capacity.

### 3.4. Synergistic Barrier Destruction by Secondary Bacterial Co-Infections

In the reality of commercial animal production, viral infections rarely occur in an ecological vacuum. While viral cytopathology often serves as the initiating event, secondary or endemic bacterial co-infections are frequently the actual drivers of sustained barrier collapse, chronic dysbiosis, and intestinal stem cell exhaustion. Once a primary viral hit—such as an acute infection by PEDV, TGEV, or Rotavirus—breaches the physical barrier, depletes the protective mucus layer, and compromises local immune surveillance, the mucosal environment becomes highly susceptible to opportunistic and enteric pathogens like *Escherichia coli* and *Salmonella* [46].

Crucially, viral injury fundamentally alters the mucosal metabolic landscape, paving the way for synergistic destruction. Under homeostatic conditions, healthy enterocytes maintain epithelial hypoxia, which suppresses the expansion of facultative anaerobic pathogens. However, the severe enterocyte necrosis induced by acute viruses like PEDV or Rotavirus, coupled with the influx of inflammatory neutrophils, disrupts colonocyte metabolism and shifts mucosal oxygenation. This increased oxygen availability—often termed the “oxygen hypothesis” of dysbiosis—fuels the rapid luminal expansion of *Salmonella* and pathogenic *E. coli* [87].

Furthermore, this altered microenvironment facilitates the intracellular proliferation of obligate intracellular bacteria, such as *Lawsonia intracellularis*. Initial crypt damage, hyperplastic responses, and disrupted cell cycle dynamics driven by primary enteric viruses or systemic viruses with enteric involvement create a highly favorable niche for *L. intracellularis* invasion into the crypt epithelium [88]. By usurping the proliferative compartment, *L. intracellularis* exacerbates the initial viral stem cell exhaustion and prevents normal epithelial restitution. Through these synergistic mechanisms, secondary bacterial infections transition an acute viral shedding event into a chronic structural failure of the barrier, characterized by sustained TJ disruption, persistent inflammatory cascades, and defective regeneration. Therefore, evaluating virus-induced barrier failure requires integrating these viral–bacterial synergies rather than viewing viral pathogenesis in isolation.

## 4. Collapse of the Mucus Barrier: The First Line of Chemical Defense

**Figure 5:**
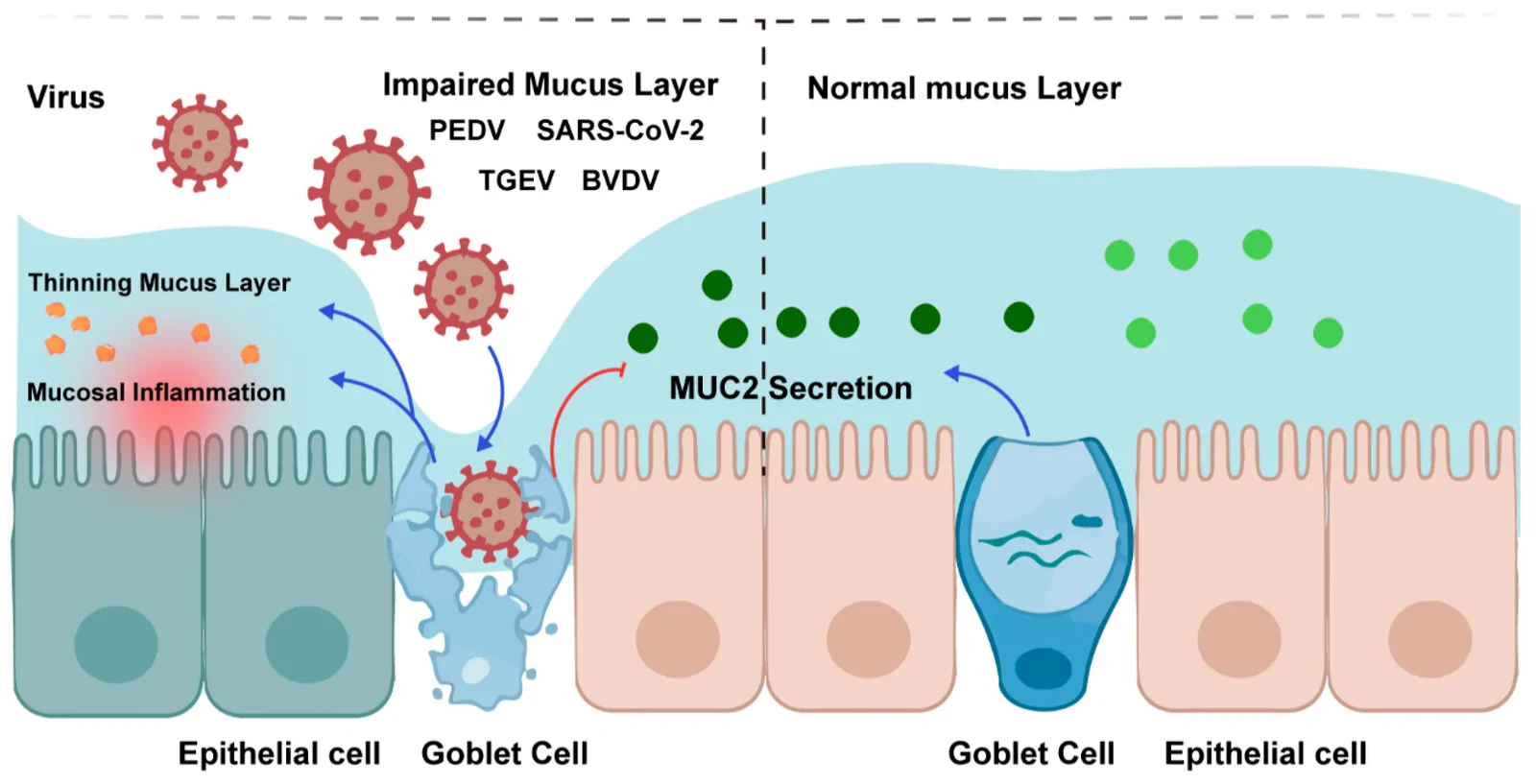
Mechanism of virus-induced disruption of the intestinal mucus barrier. Viral infections (such as PEDV, SARS-CoV-2, TGEV, and BVDV) impair goblet cell MUC2 secretion, leading to mucus layer thinning, mucosal inflammation, and compromised epithelial integrity.

The mucus barrier, primarily composed of goblet cell-derived MUC2, represents the first chemical and physical interface protecting the intestinal epithelium and limiting pathogen contact (Figure 5, Table 2). Viral infection disrupts this barrier through a multifaceted cascade involving impaired cellular differentiation, suppressed transcriptional regulation, altered post-translational modifications, and physical degradation.

Crucially, viruses fundamentally compromise mucus barrier renewal by subverting goblet cell differentiation. The continuous replenishment of goblet cells is strictly regulated by pathways such as Notch and the ATOH1/KLF4 axis. During acute viral enteritis caused by coronaviruses, elevated infection signaling activates the Notch-Hes1 pathway. This activation functions as a potent repressor of key secretory lineage regulators like ATOH1 and KLF4, thereby stalling intestinal stem cell differentiation into goblet cells and severely curtailing the cellular source of new MUC2 [89].

Beyond cellular depletion, viruses actively disrupt the intracellular synthesis and extracellular properties of the mucus. At the transcriptional level, *MUC2* expression is highly sensitive to viral infection; pathogens induce acute epithelial injury and inflammatory cascades that suppress baseline *MUC2* transcription and protein abundance [90]. Furthermore, effective barrier function relies not just on the quantity of MUC2, but on its structural integrity dictated by extensive O-glycosylation. Viral infections can alter host mucin glycoproteins and their glycan structures. These glycosylation modifications directly impact mucus rheology, shifting the mucus from a robust, viscoelastic gel to a structurally compromised state that facilitates secondary pathogen penetration [91].

This barrier disruption occurs via both direct and indirect mechanisms. Direct damage is vividly exemplified by SARS-CoV-2 and enteric viruses such as PEDV, TGEV, and BVDV, which can directly infect goblet cells, suppress MUC2 secretion, and induce local inflammatory responses, resulting in rapid mucus depletion [39,92,93,94,95,96,97,98]. Indirect disruption is prominent in systemic viral infections; both influenza virus and HIV, despite non-enteric primary tropism, can impair intestinal mucus homeostasis via sustained systemic inflammatory signaling [99,100,101]. Collectively, mucus barrier disruption represents a convergent outcome of viral infection. Key unresolved questions are whether these distinct infection modes—localized enteric viruses versus systemic viral infections—ultimately act through a shared “goblet cell–MUC2 regulatory axis” to drive mucus barrier failure, and whether mucus loss is a primary driver of epithelial injury or a secondary consequence of inflammation [102].

## 5. Immune Dysregulation as a Driver of Secondary Barrier Injury

**Figure 6:**
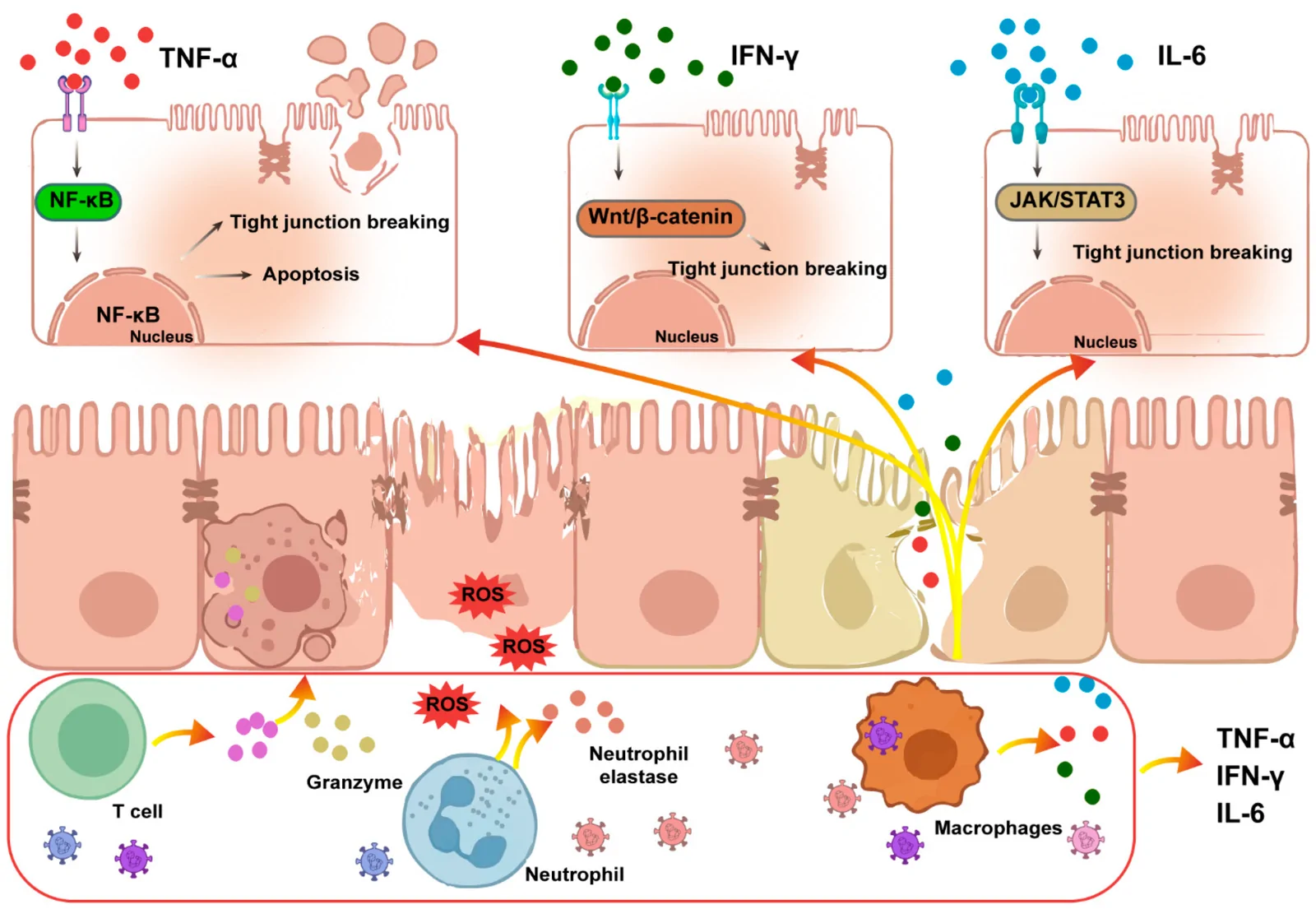
Inflammatory cytokine leading to tight junction disruption and apoptosis of epithelial cells. Viral infection activates immune cells, including T cells, neutrophils, and macrophages, resulting in the release of TNF-α, IFN-γ, and IL-6. These cytokines activate NF-κB, Wnt/β-catenin, and JAK/STAT3 signaling pathways in epithelial cells, leading to tight junction disruption and apoptosis of epithelial cells. Meanwhile, neutrophil-derived ROS and elastase amplify tissue damage, establishing an inflammatory loop that drives intestinal barrier dysfunction.

Viral infection triggers coordinated activation of innate and adaptive immune responses aimed at eliminating infected epithelial cells. However, when excessive or dysregulated, these responses shift from protective immunity to pathological inflammation, driving a secondary, immune-mediated breakdown of the intestinal barrier [103]. As illustrated in Figure 6 and Table 2, this process involves three interrelated mechanisms: cytokine-driven epithelial dysfunction, immune cell-mediated cytotoxic injury, and chronic inflammation-associated barrier failure.

### 5.1. Cytokine Signaling as a Central Driver of Epithelial Dysfunction

To understand immune-mediated barrier injury, it is necessary to separate the direct viral effects from the secondary cytokine storm. Viral infection triggers a biphasic inflammatory response with distinct cellular origins. Initially, infected epithelial cells act as the first responders, secreting chemokines and alarmins to recruit immune cells to the mucosa. Subsequently, the recruited immune cells (primarily macrophages, neutrophils, and T cells) unleash a massive secondary wave of effector cytokines, notably TNF-α, interferon-γ (IFN-γ), and interleukin-6 (IL-6) [104]. It is these high concentrations of immune-derived cytokines, rather than the virus itself, that drive this secondary epithelial dysfunction and apoptosis [105].

While direct viral infection can trigger early TJ disruption (as detailed in Section 3), sustained immune-derived cytokines act as tissue-wide stressors. By binding to TNFR1 on the epithelial surface, macrophage-derived TNF-α activates the NF-κB pathway to chronically repress ZO-1 and occludin transcription and triggers caspase-8-mediated apoptosis, expanding barrier leakage far beyond the initially infected cells [106]. T cell-derived IFN-γ acts synergistically with TNF-α by suppressing TJ protein synthesis through inhibition of the Wnt/β-catenin pathway and upregulating pro-apoptotic proteins [107]. Additionally, IL-6 contributes to progressive barrier dysfunction by activating the JAK/STAT3 pathway, altering epithelial polarity, and sustaining local inflammation [108,109]. Together, the sequential interplay between epithelial-derived recruitment signals and immune-derived effector cytokines establishes a feed-forward loop of mucosal destruction that is completely distinct from the initial viral assault.

### 5.2. Immune Cell-Mediated Cytotoxicity and Bystander Damage

Following epithelial chemokine signaling, massive numbers of immune cells are recruited to the intestinal mucosa. While this is critical for viral clearance, overactivation results in extrinsic physical destruction of the epithelium, distinct from the direct viral cytopathy discussed in Section 3 [110]. In adaptive immunity, overactivation of CD8^+^ T cells produces extensive “bystander damage,” indiscriminately killing uninfected neighboring epithelial cells via the perforin and granzyme pathways and rupturing continuous mucosal sheets [111]. Innate immune cells amplify this destruction; activated neutrophils release reactive oxygen species (ROS) and neutrophil elastase that chemically degrade TJ proteins and lipid membranes, while overactivated macrophages secrete proteases that injure the epithelial cells and mucus layer, exacerbating barrier dysfunction regardless of viral presence [112,113,114].

This immune-mediated collateral damage is widespread across viral etiologies. SARS-CoV-2 triggers overactivation of macrophages and neutrophils and abnormal proliferation of CD8^+^ T cells, causing extensive epithelial damage [115]. Rotavirus and its non-structural protein NSP4 induce CD8^+^ T cell accumulation and macrophage activation, amplifying bystander epithelial damage [116,117]. Similarly, PEDV and BVDV stimulate excessive innate immune activation and CD8^+^ T cell proliferation, leading to widespread structural necrosis and microbial translocation [118]. Furthermore, influenza viruses activate intestinal immune responses via the “gut-lung axis,” recruiting neutrophils that mechanically compromise TJs independently of direct viral replication in the gut [119]. In chronic infections like HIV, the mechanisms linking sustained CD4^+^ T cell depletion and macrophage activation to progressive barrier erosion remain poorly dissected [120]. Finally, a key unresolved question is how viral factors shape this spatial immune organization, and whether disrupted epithelial-derived chemokine gradients cause immune cells to indiscriminately target uninfected niches.

## 6. Microbiota–Virus Interplay in Barrier Destabilization

**Figure 7:**
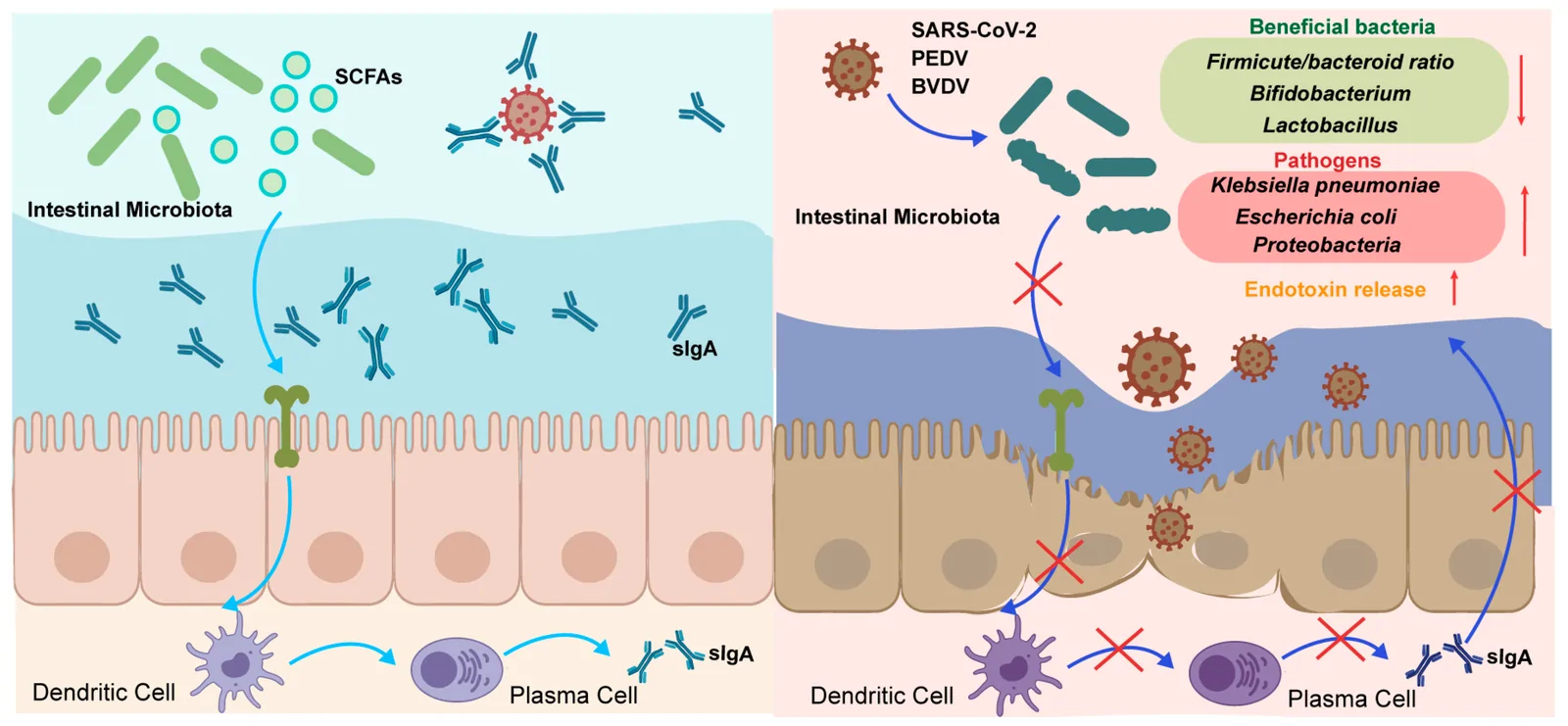
Mechanism of virus-induced disruption of the intestinal flora. Schematic of gut microbiota–immune interactions under homeostasis (**left**) and viral infection (**right**). Under physiological conditions, beneficial microbes produce short-chain fatty acids (SCFAs) and support dendritic cell-mediated induction of plasma cells and secretion of secretory IgA (sIgA), maintaining barrier integrity and immune balance. Viral infection (such as SARS-CoV-2, PEDV, and BVDV) induces microbial dysbiosis, characterized by loss of beneficial bacteria and expansion of pathogenic taxa, alongside reduced sIgA production. These changes weaken mucosal defense, promote microbial translocation and endotoxin release, and amplify inflammation, ultimately driving barrier dysfunction.

The gut microbiota represents a dynamic ecological system essential for maintaining intestinal barrier integrity and host immune homeostasis. Viral infection and the microbiota engage in a tightly interconnected bidirectional network, in which each component shapes the composition and function of the other, ultimately determining barrier integrity and disease outcome [121,122] (Figure 7, Table 2).

### 6.1. Virus-Driven Ecological Remodeling of the Gut Microbiota

When assessing flora–virus interactions, the large-scale ecological shifts observed during acute infection primarily represent downstream consequences of virus-induced host responses rather than the initiating events. Under normal conditions, the microbiota is dominated by Firmicutes and Bacteroidetes [123,124,125]. However, virus-induced epithelial damage and subsequent inflammation cause host cells to release reactive oxygen species (ROS), antimicrobial peptides, and inflammatory cytokines. This altered mucosal microenvironment—characterized by increased oxygen tension and nutrient reprogramming—selectively eradicates oxygen-sensitive obligate anaerobes (such as Firmicutes) and creates a niche for oxygen-tolerant facultative anaerobes to thrive [126,127].

This cascade explains the widespread dysbiosis observed across diverse viral infections. For instance, HIV and SARS-CoV-2 infections trigger mucosal inflammation that leads to a profound depletion of beneficial bacteria (e.g., *Bifidobacterium* and *Lactobacillus*) and the enrichment of opportunistic pathogens (e.g., *Klebsiella pneumoniae* and *Escherichia coli*), with dysbiosis severity directly correlating with the host’s inflammatory response [128,129,130,131]. Even respiratory viruses like influenza indirectly induce gut dysbiosis via systemic immune responses, while enteric viruses (rotavirus, norovirus, PEDV, BVDV) consistently promote pathogenic bacterial overgrowth due to direct epithelial lysis [39,132,133,134,135,136,137]. Virus-mediated immune dysregulation further weakens host control over microbial homeostasis, perpetuating this ecological collapse [103]. Ultimately, this resultant dysbiosis compromises the microbial barrier, facilitating bacterial translocation and endotoxin release, which further amplify mucosal injury [130,138].

### 6.2. Microbiota Control of Viral Susceptibility and Replication

Conversely, specific pre-existing microbial taxa and dysbiotic states act as direct causal factors that dictate viral infectivity and replication efficiency [125,139]. In this context, bacteria function not as passive bystanders, but as active cofactors or inhibitors of viral pathogenesis. On one hand, commensal bacteria act as causal agents of antiviral defense by continuously priming innate and adaptive immunity [140,141]. Beneficial microbes such as *Bifidobacterium* and *Lactobacillus* promote dendritic cell maturation and stimulate secretory IgA (sIgA) production via TLR2/TLR4 signaling pathways, thereby strengthening mucosal immunity and limiting viral infection and replication, including rotavirus and norovirus [134,142].

On the other hand, dysbiosis-associated pathogenic bacteria can facilitate viral infection. Certain pathogens suppress host immune responses or provide binding sites that enhance viral adhesion and transmission [143,144,145]. For example, commensal bacteria such as *Enterobacter cloacae* express histo-blood group antigen (HBGA)-like carbohydrates on their surface; these bacterial HBGAs directly bind to human norovirus particles, acting as essential cofactors that transport the virus to the epithelial surface and protect it from environmental stressors, thereby directly causing enhanced viral infectivity [146]. Additionally, pathogens belonging to the *Enterobacteriaceae* family can produce metabolites that actively inhibit sIgA secretion, impairing mucosal immune exclusion and promoting rotavirus infection [147]. These mechanisms demonstrate that specific flora directly govern viral entry at the level of receptor accessibility and immune evasion.

### 6.3. Metabolic Mediators Linking Microbiota to Barrier Integrity

The gut microbiota is a key regulator of intestinal barrier integrity during viral infection, and its homeostasis is strictly surveilled by the host’s chemical defenses. AMPs, primarily secreted by Paneth cells in the small intestine, play a dual role in maintaining intestinal homeostasis and providing direct antiviral defense. Mechanistically, AMPs such as defensins can directly neutralize viruses or disrupt their lipid envelopes, effectively blocking the cellular entry of pathogens like HIV [148]. In response, viruses have evolved sophisticated mechanisms to evade these defenses; for example, HIV and certain enteric viruses can downregulate the host expression of specific AMPs to facilitate their replication and dissemination [149]. Crucially, the profound impact of viruses on gut microbiota remodeling frequently results from virus-driven damage to Paneth cells. Viral infections, such as those caused by SARS-CoV-2, can directly infect or induce apoptosis in Paneth cells, drastically reducing the secretion of AMPs [150]. Dysbiosis can directly impair the microbial barrier and disrupt the coordinated function of physical, chemical, and immune barriers, thereby exacerbating epithelial damage [151], largely due to the loss of beneficial bacteria and the resulting deficit in critical metabolites like short-chain fatty acids (SCFAs). SCFAs—particularly butyrate and propionate—are critical, active causal mediators of TJ assembly and maintenance [152,153]. Rather than merely serving as an energy source for colonocytes, SCFAs dictate barrier integrity through specific molecular pathways. One major mechanism is G-protein-coupled receptor (GPCR) signaling; butyrate and propionate act as direct ligands for specific receptors (e.g., GPR41, GPR43) on the intestinal epithelium. Receptor activation triggers the AMP-activated protein kinase (AMPK) pathway, which is strictly required for the dynamic reorganization of the actin cytoskeleton and the proper apical-basolateral assembly of the ZO-1 and occludin networks, thereby physically tightening the paracellular space [154]. In parallel, SCFAs dictate barrier function through epigenetic regulation via histone deacetylase (HDAC) inhibition. Butyrate functions as a potent endogenous inhibitor of HDACs, promoting the hyperacetylation of histones at the promoter regions of TJ genes. This epigenetic modification directly upregulates the transcription and subsequent protein expression of critical barrier components, including Claudin-1 and Occludin [155]. Unfortunately, viral infection-induced dysbiosis rapidly depletes SCFA-producing bacteria, causing a dramatic reduction in luminal butyrate and propionate [156]. The loss of SCFAs immediately removes both the AMPK-mediated structural stabilization and the HDAC-mediated transcriptional upregulation of TJs, rendering the epithelial barrier highly susceptible to virus-induced physical breakdown and apoptosis. Consequently, restoring microbial homeostasis and SCFA levels represents a direct, pathway-specific strategy to fortify the intestinal barrier against viral assault.

**Table 2:** Viral infection induced intestinal barrier dysfunction.

Barrier	Mechanism Module		Key Pathways/Molecules	Representative Viruses	Main Outcome	References
Physical Barrier	Tight junction		Occludin, claudins, ZO-1/ZO-2; NF-κB/MLCK; RhoA/ROCK; PI3K/Akt; ubiquitin ligases (Itch),	TGEV, Norovirus, PEDV, Rotavirus, SARS-CoV-2, WNV, ZIKV, BVDV, H5N1, ASFV	Increased epithelial permeability, TJ disassembly	[34,35,36,39,40,42,43,44,45]
	Intestinal stem cell		Wnt/β-catenin pathway, cell cycle regulators	SARS-CoV-2, Rotavirus, PEDV, TGEV	Impaired intestinal stem cell renews	[46,82,83,85,86]
	Epithelial cell	Apoptosis	Caspase-8/9/3/7, Fas/FasL	HIV, Rotavirus, Norovirus, TGEV, PEDV, SARS-CoV-2, CPV-2	Epithelial cell death	[57,58,59,60,61,62,63]
		Pyroptosis	NLRP3, Caspase-1, GSDMD	SARS-CoV-2, IAV, RSV, Adenovirus	Strong inflammation	[64,65]
		Necroptosis	RIPK3/MLKL	SARS-CoV-2, BVDV	Inflammation and epithelial cell death	[39,40]
		Autophagy	PI3K/AKT/mTOR, ATG5/12, Beclin-1	Norovirus, BVDV, SARS-CoV-2, HCV	Epithelial cell death	[67,68,69,70]
		Ferroptosis	GPX4, ROS, iron overload	SARS-CoV-2, Influenza virus	Epithelial cell death	[73,74,75,76]
		Cuproptosis	CTR1, ATP7A/B, FDX1	SARS-CoV-2	Epithelial cell death	[78,79]
Chemical Barrier	Mucus barrier		MUC2, inflammatory cytokines, systemic immune signaling	SARS-CoV-2, Influenza virus, HIV, PEDV, TGEV, BVDV	Thinning of mucus layer, pathogen exposure	[39,92,93,94,95,96,97,98,102]
Immune Barrier	Cytokine		NF-κB, JAK/STAT3, Wnt inhibition	SARS-CoV-2 and others	Strong inflammation	[108,109]
	Immune cell		Perforin/granzyme, ROS, elastase	SARS-CoV-2, Rotavirus, PEDV, BVDV, Influenza	Strong inflammation	[115,116,117,118,119,120]
	Persistent inflammatory damage		NF-κB pathway	HIV, chronic viral infections	Strong inflammation	[120]
Microbial Barrier	Gut microbiota dysbiosis		Immune dysregulation, metabolic imbalance	HIV, SARS-CoV-2, Influenza, Rotavirus, Norovirus, PEDV, BVDV	Microbial barrier collapse	[39,128,129,130,131,134,135,136,137]

Mechanisms of viral infection-induced intestinal barrier dysfunction.

## 7. Therapeutic Strategies: From Viral Control to Barrier Restoration

Virus-induced disruption of the intestinal barrier represents a central pathogenic axis in viral infectious diseases. Accordingly, therapeutic strategies increasingly focus on combining antiviral clearance with barrier restoration to interrupt the progression of viral infection–barrier damage–systemic disease [157]. Importantly, treatment selection must be tailored to the virus’s specific pathogenic mechanism: enterogenous viruses (e.g., rotavirus and norovirus) primarily require local mucosal protection and direct antiviral control, whereas systemic viruses (e.g., HIV and SARS-CoV-2) require strategies that address profound systemic immune dysregulation and secondary barrier damage. Crucially, in the context of veterinary sciences, these interventions must be adapted to the realities of herd health and population medicine. The therapeutic focus in commercial animal production must pivot toward economically viable strategies against high-impact pathogens like PEDV, TGEV, and BVDV. Current approaches can be broadly categorized into antiviral therapies, barrier-protective interventions, microbiota-based strategies, and emerging regenerative modalities (Table 3).

### 7.1. Antiviral Therapies

Vaccination remains the most effective preventive strategy, inducing virus-specific humoral and cellular immunity to block infection and preserve epithelial integrity [158]. Licensed vaccines against rotavirus, influenza, and SARS-CoV-2 have substantially reduced disease burden, particularly the incidence of virus-associated intestinal injury [159]. However, in veterinary population medicine, because conventional injectable vaccines often fail to elicit robust secretory IgA (sIgA) responses in the gut, the focus has pivoted to mucosal vaccine platforms (oral and intranasal) for swine and poultry. Furthermore, leveraging maternal lactogenic immunity—such as vaccinating pregnant sows to deliver protective sIgA to neonatal piglets via colostrum—is the most effective strategy for preventing acute enterocyte necrosis caused by PEDV [160]. Next-generation platforms, including mRNA and viral vector vaccines, aim to broaden coverage against rapidly evolving variants [161].

For established infection, antiviral drugs are essential, but their application varies by viral tropism. These agents primarily inhibit viral replication by targeting key enzymatic processes. Antiviral drugs against SARS-CoV-2, such as nirmatrelvir/ritonavir, can block viral replication by inhibiting the virus’s 3CL protease [162], while antiretroviral therapy (ART) effectively limits HIV replication and indirectly protects intestinal barrier integrity [163]. Although candidates such as molnupiravir and polymerase inhibitors show activity against rotavirus and norovirus in experimental systems [164,165], clinically approved therapies for most enteric viruses remain unavailable. Protease-targeting compounds such as GC376 and PF-00835231 further demonstrate broad activity against coronaviruses including PEDV and TGEV [166,167], though they remain experimental. Furthermore, a major limitation across all viral types is that antivirals primarily suppress replication without reversing established epithelial injury or restoring barrier architecture. Thus, antiviral therapy alone is insufficient for full mucosal recovery.

### 7.2. Barrier-Protective Therapies

Barrier-protective strategies aim to restore epithelial integrity and suppress inflammatory amplification through coordinated targeting of TJs, immune signaling, and mucosal defense [168]. However, the quality of clinical evidence for many of these agents is currently low, and their efficacy should not be overestimated. In commercial production, the economic viability of delivering these stabilizers as feed additives is paramount. Glutamine enhances ZO-1 and occludin expression and supports epithelial repair by providing essential metabolic fuel for rapid enterocyte turnover during viral enteritis [169,170,171], while natural compounds such as baicalin and curcumin promote junctional reassembly and reduce epithelial permeability [172]. Additionally, trace minerals like zinc oxide have historically been utilized in swine diets to tighten mucosal junctions and reduce permeability [173]. However, their efficacy is limited under conditions of active viral replication or sustained inflammation, and bioavailability constraints reduce clinical consistency.

Anti-inflammatory therapies, including glucocorticoids and cytokine-targeting antibodies, suppress NF-κB-driven inflammatory cascades and mitigate cytokine-mediated barrier injury [174,175,176,177]. While effective in conditions such as severe COVID-19 and HIV-associated inflammation, their use is constrained by systemic immunosuppression, impaired antiviral clearance, and metabolic side effects. Mucosal protectants such as montmorillonite and sucralfate provide a physical barrier at the luminal surface while enhancing MUC2-mediated mucus production [178,179]. These agents reduce pathogen–epithelial contact but do not address deeper epithelial or stem cell damage, limiting their standalone efficacy.

### 7.3. Microbiota-Based Therapies

Microbiota-targeted interventions represent an emerging adjunctive strategy to restore microbial homeostasis and reinforce barrier function [180,181,182], For acute enterogenous viral infections (e.g., rotavirus and norovirus), probiotics (e.g., *Lactobacillus rhamnosus GG* and *Saccharomyces boulardii*) have moderate- to high-quality clinical evidence for shortening the duration of diarrhea through competitive exclusion and short-chain fatty acid (SCFA) production [183,184,185]. Clinical studies support their efficacy in reducing viral diarrhea and improving outcomes in COVID-19 patients [186,187]. Prebiotics (e.g., inulin and oligosaccharides) selectively promote beneficial bacterial growth and enhance SCFA production, providing a stable metabolic substrate for barrier protection [188]. Conversely, for systemic viral infections (e.g., HIV and SARS-CoV-2), probiotics serve more as systemic immunomodulators rather than direct antiviral agents. In these severe systemic contexts, Fecal Microbiota Transplantation (FMT) is being explored to correct profound, long-term dysbiosis and immune depletion [189,190,191,192]. While FMT is rarely indicated for acute enterogenous viruses (unless complicated by *C. difficile*), it shows promising proof of concept for HIV-associated enteropathy and COVID-19. However, in veterinary medicine, the application of FMT in commercial livestock is severely limited by biosecurity risks and unstandardized protocols. Therefore, the veterinary industry relies primarily on scalable synbiotic blends to achieve herd-level microbiota modulation, particularly during the highly vulnerable post-weaning window [193]. However, its use remains limited by donor variability, safety concerns, and unstandardized protocols. Ultimately, microbiota modulation cannot overcome ongoing, unsuppressed viral replication, making it strictly an adjunctive therapy.

### 7.4. Emerging Therapeutic Approaches

Advances in virus–host biology have enabled novel regenerative strategies targeting epithelial repair and host resistance. Gene therapy approaches, including CRISPR-Cas9-based editing, primarily focus on systemic viruses by targeting specific viral entry receptors (e.g., knocking down ACE2 to block SARS-CoV-2) or attempting to upregulate TJ proteins [194,195]. For high-impact agricultural pathogens, notable proof-of-concept successes include the development of CRISPR-engineered pigs with Aminopeptidase N (ANPEP) knockouts, which demonstrate innate genetic resistance to coronaviruses like TGEV and PEDV [196]. Despite strong mechanistic potential, clinical translation is limited by delivery, safety, and ethical constraints. Cell-based therapies, particularly intestinal stem cell and mesenchymal stem cell transplantation, promote mucosal regeneration via cytokine secretion, immunomodulation, and epithelial repair [197,198,199,200]. This approach is highly unlikely to be necessary for self-limiting enterogenous viruses, which usually preserve native stem cell niches. Instead, stem cell therapy is specifically positioned for catastrophic barrier failure or chronic mucosal atrophy caused by severe systemic immune responses (e.g., COVID-19 cytokine storm or end-stage HIV enteropathy). Nevertheless, challenges remain regarding engraftment efficiency, functional integration, and long-term stability of regenerated epithelium.

**Table 3:** Therapeutic strategies targeting virus-induced intestinal barrier dysfunction.

Therapeutic Category	Subtype	Representative Agents/Examples	References
Antiviral	Vaccines (preventive strategy)	Rotavirus vaccine, influenza vaccine, SARS-CoV-2 vaccine; next-generation platforms (mRNA vaccines, viral vector vaccines)	[159,161]
	Antiviral drugs (post-infection therapy)	SARS-CoV-2: nirmatrelvir/ritonavir (3CLpro inhibitor); HIV: ART (reverse transcriptase/protease inhibitors); Rotavirus/Norovirus: Molnupiravir; PEDV: GC376 and derivatives; PF-00835231 (broad-spectrum 3CLpro inhibitor targeting PEDV, TGEV, SADS-CoV)	[162,163,164,165,166,167]
Intestinal barrier protection therapy	Tight junction stabilizers	Glutamine; baicalin; curcumin	[170,171,172]
	Anti-inflammatory agents	Glucocorticoids; anti-TNF-α antibodies; anti-IL-6 antibodies; anti-inflammatory cytokines	[175,176,177]
	Mucosal protectants	Montmorillonite powder; sucralfate	[178,179]
Gut microbiota-based therapy	Probiotics	Bifidobacteria, Lactobacillus, Saccharomyces boulardii	[186,201,202,203,204]
	Prebiotics	Fructooligosaccharides, galacto-oligo saccharides, inulin	[187,188]
	Fecal microbiota transplantation (FMT)	Healthy donor fecal microbiota	[190]

## 8. Future Perspectives

While significant strides have been made in characterizing virus-induced intestinal barrier injury, advancing the field requires moving beyond descriptive summaries of acute damage toward testing verifiable, long-term mechanistic hypotheses. A critical, yet underexplored frontier is the concept of virus-induced “epigenetic memory” within the intestinal stem cell (ISC) niche. We hypothesize that transient viral infections may induce lasting epigenetic scars in ISCs and their supporting mesenchymal cells, fundamentally altering steady-state epithelial regeneration and predisposing hosts to chronic post-viral barrier dysfunction. Investigating whether highly specific, localized virus–host interactions permanently reprogram these regulatory networks—rather than merely triggering transient acute cascades—represents a crucial new direction for the field. Crucially, translating these molecular insights into effective therapeutics requires abandoning homogenous experimental models to address the realities of human diversity. Future investigations must explicitly interrogate how genetic background (e.g., polymorphisms in innate immune sensors or TJ architecture) dictates individual susceptibility to barrier failure. Furthermore, the influences of sex and age remain glaringly understudied despite being undeniable factors in clinical outcomes. It is imperative to define how sex-specific hormonal landscapes and X-linked immune regulators modulate epithelial resilience, and how age-related variations—from pediatric mucosal immaturity to geriatric ISC senescence—dictate the trajectory of barrier repair. Rather than generically applying spatial or single-cell omics as a standard pipeline, the field must strategically deploy these technologies on demographically diverse, patient-derived enteroid biobanks. By mapping the spatiotemporal dynamics of viral injury across these distinct demographic and genetic variables, researchers can identify personalized, actionable targets that reflect the true clinical complexity of viral enteropathies.

## 9. Conclusions

Virus-induced intestinal barrier disruption represents a central pathogenic axis linking localized infection to systemic disease. Across diverse viruses, distinct mechanisms—including junctional disassembly, epithelial cell death, immune dysregulation, and microbiota remodeling—converge on a shared outcome: collapse of barrier integrity through interconnected and self-amplifying processes. This systems-level failure promotes viral dissemination, inflammation, and multi-organ pathology. Despite recent advances, key mechanistic gaps remain. In particular, the regulatory nodes integrating epithelial, immune, and microbial signals are poorly defined, and the failure of barrier repair—especially involving intestinal stem cells and niche dynamics—remains largely unexplored. The causal role of microbiota alterations in disease progression also requires clarification. Future progress will depend on spatiotemporally resolved, systems-level approaches to disentangle primary viral effects from host-driven responses and to identify actionable targets. Therapeutically, these insights highlight the need to move beyond antiviral strategies toward restoring barrier integrity and mucosal homeostasis. Together, positioning the intestinal barrier as an active determinant of disease outcome provides a unifying framework for understanding viral pathogenesis and developing next-generation interventions.

## Data Availability

No new data were created or analyzed in this study. Data sharing is not applicable to this article.
